# *PsAAT3* Drives Ester Accumulation and Fruity Aroma Formation During Ripening in Chinese Plum (*Prunus salicina*) Through Integrated Volatile Profiling and Transcriptomics

**DOI:** 10.3390/plants15081144

**Published:** 2026-04-08

**Authors:** Wenqian Zhao, Sujuan Liu, Siyu Li, Gaigai Du, Longji Li, Danfeng Bai, Gaopu Zhu, Shaobin Yang, Fangdong Li, Taishan Li, Haifang Hu

**Affiliations:** 1 College of Horticulture and Forestry, Tarim University, Alar 843300, China; finiea9410@sina.cn; 2 Key Laboratory of State Forestry and Grassland Administration on Desert Oasis Ecosystem Protection and Restoration, Urumqi 830000, China; 3 Jiamu National Long-Term Scientific Research Base for Pomology, Aksu 843101, China; 4 Research Institute of Non-Timber Forestry, Chinese Academy of Forestry, Zhengzhou 450003, China; sujuanliu07@163.com (S.L.); lxlisiyu@163.com (S.L.); gaigaidu@126.com (G.D.); 12456agdf@gmail.com (L.L.); baidanfeng1993@163.com (D.B.); zhugaopu@163.com (G.Z.); ysb1966327@aliyun.com (S.Y.); lifangdong66@163.com (F.L.); 5 Xinjiang Academy of Forestry Science, Urumqi 830000, China

**Keywords:** esters, flavor attribute, *PsAAT3*, ripening stage, VOCs, WGCNA

## Abstract

Fruit volatile organic compounds (VOCs) are key determinants of plum flavor quality, and esters contribute strongly to the fruity aroma of ripe fruit. However, the molecular basis of cultivar differences in ester formation during ripening has not been systematically clarified. Here, we characterized pulp VOC profiles across ripening in three Chinese plum (*Prunus salicina*) cultivars (‘WeiWang’ (WW), ‘WeiDi’ (WD), and ‘KongLongDan’ (KLD)) and integrated transcriptome analysis with weighted gene co-expression network analysis (WGCNA) to identify genes associated with ester accumulation. HS-SPME-GC-MS identified 38 VOCs, mainly esters, aldehydes, and alcohols, with ‘WW’ showing the highest total VOC abundance. During ripening, esters became the predominant volatile class in ‘WW’ and ‘WD’, in agreement with their fruity sensory characteristics, whereas ‘KLD’ maintained a more balanced composition of fruity and green-related volatiles. Transcriptomic analyses highlighted *Prunus salicina* alcohol acyltransferase 3 (*PsAAT3*) as the most abundant AAT transcript in pulp and strongly induced in ‘WW’. Transient overexpression of *PsAAT3* in the low-ester background increased butyl acetate and hexyl acetate by 4.8- and 2.2-fold, respectively. WGCNA further identified ester-associated modules and candidate transcription factors co-expressed with *PsAAT3* (*JA2L*, *HY5*, *NAC073*, and *PHL13*). As a result, this study identifies *PsAAT3* as a key determinant of high-ester aroma in Chinese plum and provide candidate targets for aroma improvement and flavor-oriented breeding.

## 1. Introduction

In recent years, fruit aroma has attracted increasing attention as a key sensory trait and has become an important determinant of fruit quality and consumer acceptance [1,2,3,4]. Aroma formation relies on dynamic changes in volatile organic compounds (VOCs) during fruit development and ripening. The major VOC classes include esters, aldehydes, alcohols, terpenes, lactones, and ketones [5,6]. Different classes of VOCs contribute in different ways to perceived aroma. Esters are most often linked to sweet, fruity notes; aldehydes and alcohols tend to impart green notes; and terpenes together with lactones are frequently associated with floral, honey-like, or creamy characters [7]. In many fruits, esters contribute most of the aroma at ripening. For example, in two ripe ‘Binzi’ apple cultivars, esters account for about 56% and 52% of the total volatiles, making them the most abundant VOCs [8]. Similarly, in strawberries and pears, esters are also key abundant compounds during ripening and generally contribute to fruity and floral notes [9,10]. Furthermore, when ester compounds were removed from ‘Fuji’ apples, the intensity of the fruity aroma dropped by over 90%. This finding highlights the central role of esters in fruit aroma formation [11]. Given their significant impact on fruit aroma, it is important to understand how esters are produced.

Ester formation involves several metabolic pathways, including fatty acid metabolism, branched-chain amino acid metabolism, and β-oxidation [12]. Among these, the fatty acid pathway provides key precursors and supports the key esterification step, and it is therefore considered a major pathway for ester biosynthesis [13]. In this pathway, lipoxygenase (LOX) oxidizes unsaturated fatty acids and produces hydroperoxides. hydroperoxide lyase (HPL) then cleaves these intermediates to generate C6/C9 aldehydes. alcohol dehydrogenase (ADH) converts aldehydes to the corresponding alcohols. alcohol acyltransferase (AAT) transfers acyl groups from acyl-CoA donors to alcohols and forms volatile esters [14]. Thus, LOX, HPL, and ADH influence the formation of aldehyde and alcohol precursors, whereas AAT catalyzes the final step of ester production. For instance, in apples, upregulation of *MdLOX1a* increases fatty acid-derived volatiles [15]. In peach, downregulation of *PpHPL1*, *PpADH1*, *PpADH2*, and *PpADH3* during ripening reduces C6 alcohols, which further affects substrate supply for ester formation [14]. AAT is widely considered one of the key enzymes controlling ester synthesis. Its expression level is closely linked to the accumulation of esters in fruit. It has been reported that overexpression of *AAT1* in apricots and pears increases ester production [16,17]. In addition, fatty acid desaturases can influence ester formation by altering precursor availability, whereas carboxylesterases can lower ester levels through hydrolysis [18,19]. At the transcriptional level, multiple transcription factors (TFs) have been shown to regulate ester biosynthesis-related genes, particularly AATs [5]. For example, in peach (Prunus persica), apple, and pear, transcription factors such as PpNAC1 together with PpDML1, MdMYB85, and the PcbZIP44–PcWRKY70 complex have been implicated in the transcriptional activation of AAT genes during fruit ripening [6,17,20,21]. Together, these studies indicate that both structural genes and their upstream TF regulators are important determinants of ester-derived aroma.

Chinese plum (*Prunus salicina*) is a Rosaceae fruit tree species in the genus *Prunus*, and its fruits are valued for their attractive color, juicy texture, and appealing aroma. Previous studies have shown that Chinese plum fruits contain diverse volatile compounds that contribute mainly to fruity, floral, and green aroma notes, with esters being widely recognized as key contributors to their fruity aroma [22,23,24]. However, studies on plum aroma metabolism and its regulatory networks are still limited. In particular, the key biosynthetic genes and upstream regulatory factors responsible for differences in ester accumulation among Chinese plum cultivars during ripening are still not well understood [25,26]. We hypothesized that fruity Chinese plum cultivars differ in their main volatile classes, especially in the composition and levels of esters. These differences are likely related to changes in the expression of key genes in the LOX pathway, particularly AAT genes and their upstream transcription factors. To test this, we selected three Chinese plum cultivars with clear differences in fruity aroma for this study. We measured pulp VOCs at different ripening stages and focused on ester accumulation and related sensory changes. We identified key genes in the LOX pathway through orthogroup analysis and examined their expression patterns using RNA sequencing (RNA-seq) data. We further applied Weighted gene co-expression network analysis (WGCNA) to identify gene modules associated with ester accumulation and to screen candidate transcription factors involved in aroma formation. These results provide a framework for understanding the molecular basis of cultivar differences in ester production and facilitate aroma improvement and flavor-oriented breeding.

## 2. Results

### 2.1. Dynamic Changes in VOC Profiles During Ripening Among Three Plum Cultivars

**Figure 1:**
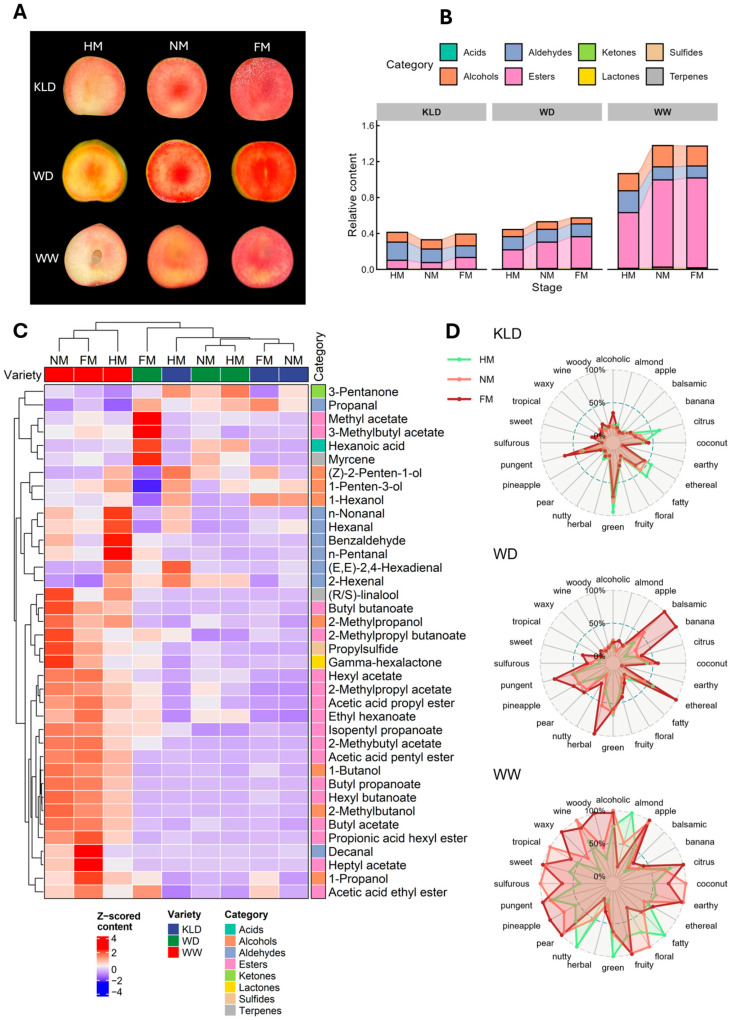
Volatile organic compound (VOC) profiles in pulps of three plum cultivars across ripening stages (‘KLD’, ‘WD’, and ‘WW’ indicate ‘KongLongDan’, ‘WeiDi’, and ‘WeiWang’). (**A**) Representative fruit characteristics at the half-mature (HM), near-mature (NM), and full-mature (FM) stages. (**B**) Changes in the relative abundance of major VOC classes across ripening stages. (**C**) Heatmap of individual VOCs with hierarchical clustering across samples. The abundance of each compound was Z-score (Standard Score) normalized across all samples (row-wise scaling). Red indicates higher relative abundance, and blue indicates lower relative abundance. (**D**) Aroma descriptor profiles of VOCs across ripening stages for each cultivar. Each axis represents one aroma category. The radius on each axis indicates its relative intensity. Lines closer to the outer circle indicate stronger aroma. The outer circle marks the maximum value for that category across all samples. Colored lines represent different ripening stages.

We analyzed volatile organic compounds (VOCs) in pulp at three ripening stages (half-mature (HM), near-mature (NM), and full-mature (FM)) from three Chinese plum (*Prunus salicina*) cultivars with fruity aroma: ‘KLD’, ‘WD’, and ‘WW’ (Figure 1A). Overall, 38 VOCs were detected, comprising 17 esters, 8 aldehydes, 7 alcohols, 2 terpenoids, and one compound each classified as a lactone, ketone, acid, and sulfide (Figure 1B, Table S1 and Figure S3). Among cultivars, ‘WW’ showed the highest total VOC abundance, while ‘KLD’ and ‘WD’ had similar total VOC levels. In all three cultivars, esters, aldehydes, and alcohols were the top three VOC groups, and the other groups accounted for smaller proportions. In ‘WW’ and ‘WD’, esters were the most abundant class. Ester levels increased as ripening progressed, and the largest increase occurred from HM to NM. At the same ripening stage, total ester abundance in ‘WW’ was 2.85–3.31-fold that in ‘WD’. In contrast, ester levels in ‘KLD’ decreased slightly at NM and then increased again at FM. In all samples, hexyl acetate, butyl acetate, and ethyl hexanoate were the three most abundant esters (Table S1). For aldehydes, total abundance generally decreased during ripening in ‘KLD’ and ‘WW’, while ‘WD’ showed no clear stage-dependent change.

Figure 1C shows the individual compound accumulations across cultivars and stages. For esters, only methyl acetate and 3-methylbutyl acetate reached their highest relative abundance in ‘WD’ pulp at FM. Most other esters were higher in ‘WW’, especially at NM and FM. This pattern supports the high-ester aroma type of ‘WW’. Aldehydes also showed clear differences among cultivars. Propanal stayed at the lowest relative level in ‘WW’. Decanal was higher in ‘WW’ and increased with ripening. N-nonanal, hexanal, benzaldehyde, and n-pentanal were higher at HM in ‘WW’ and then declined at later stages. In ‘KLD’, (E,E)-2,4-hexadienal and 2-hexenal peaked at HM and were the highest among all samples, then decreased during ripening. These declining aldehydes may relate to the gradual loss of green notes as the fruit matures. Alcohols also differed among cultivars. Some alcohols were higher in ‘KLD’, while others were higher in ‘WW’ (Figure 1C).

Different VOCs contribute to different aroma notes. We used a VOC flavor database to assign aroma categories and to estimate the relative intensity of each category in each sample (Figure 1D). ‘KLD’ showed a stronger green note at HM. ‘WD’ showed higher herbal, balsamic, ethereal, and banana-like notes at FM. ‘WW’ showed more dominant flavor categories than the other two cultivars and its overall flavor intensity at NM and FM was clearly stronger than at HM. During ripening, ‘WW’ lost much of the almond, green, fatty, and nutty notes that were more obvious at HM. At the same time, fruity notes such as apple, pear, pineapple, sweet, and fruity became stronger (Figure 1D). Across cultivars, both ‘WD’ and ‘WW’ became more fruity as ripening progressed. ‘KLD’ stayed more balanced between green and fruity notes, and its green note declined only slightly (Figure S1).

### 2.2. Ripening Effects on Key Volatiles and Cultivar-Specific Responses

**Figure 2:**
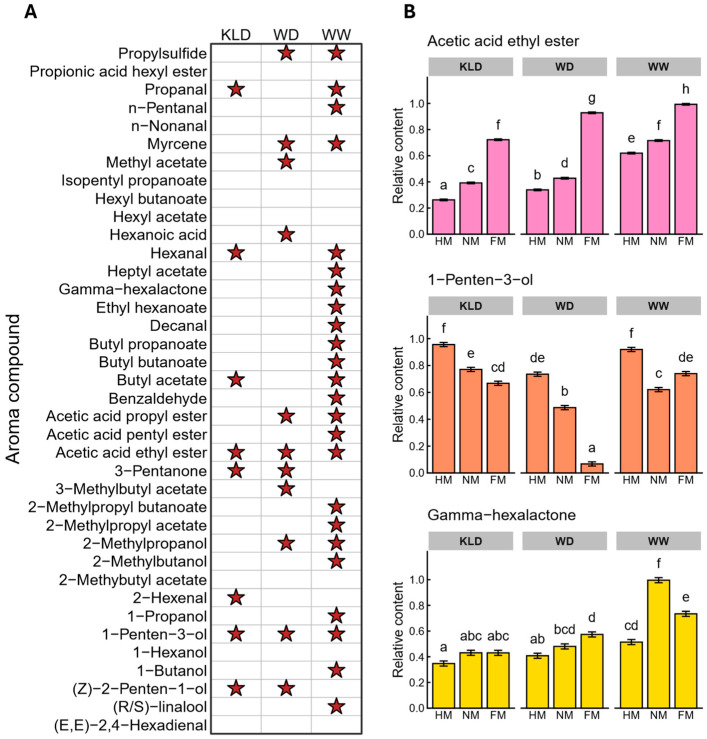
Dynamic changes in volatile organic compounds (VOCs) during ripening in three plum cultivars (‘KLD’, ‘WD’, and ‘WW’ indicate ‘KongLongDan’, ‘WeiDi’, and ‘WeiWang’). (**A**) Stage-dependent differences in the abundance of individual VOCs within each cultivar across the half-mature (HM), near-mature (NM), and full-mature (FM) stages. Red stars indicate significant differences among ripening stages within the cultivar (*p* < 0.05). (**B**) Relative abundance changes in representative VOCs (acetic acid ethyl ester, 1-penten-3-ol, and γ-hexalactone) across ripening stages in the three cultivars. Data are presented as mean ± SD (standard deviation). Different letters indicate significant differences among ripening stages within the same cultivar (*p* < 0.05), as determined by one-way ANOVA followed by Tukey’s HSD post hoc test.

Fruit ripening has a strong effect on the accumulation of VOCs [27]. In this study, we tested the relative abundance of each VOC within each cultivar across three ripening stages (Figure 2A). ‘WW’ showed significant stage differences for 24 VOCs. This number was clearly higher than that in the other two cultivars. This result suggests that aroma-related metabolism in ‘WW’ responds more strongly to ripening. Acetic acid ethyl ester and 1-penten-3-ol changed significantly with ripening in all three cultivars. Acetic acid ethyl ester increased as ripening progressed in each cultivar. The increase was more obvious from NM to FM. In contrast, 1-penten-3-ol decreased with ripening in ‘KLD’ and ‘WD’. ‘WD’ showed the strongest decrease from NM to FM. ‘WW’ showed a different pattern. In ‘WW’, 1-penten-3-ol dropped sharply at NM and then rose slightly at FM. This pattern suggests that the conversion of related precursors and the metabolic flow for this compound may differ among cultivars during late ripening. In addition, γ-hexalactone, a representative lactone often associated with ripening-related sweet and creamy or coconut-like notes [28], showed significant stage-dependent variation exclusively in ‘WW’ (Figure 2B). Its abundance increased markedly at NM and then decreased rapidly at FM, mirroring the dynamic shifts in the corresponding sensory attributes observed in the flavor radar plot for ‘WW’ (Figure 1D).

### 2.3. Ortholog Identification and Genomic Features of Key Enzyme Genes in Fatty Acid-Derived VOC Biosynthesis

**Figure 3:**
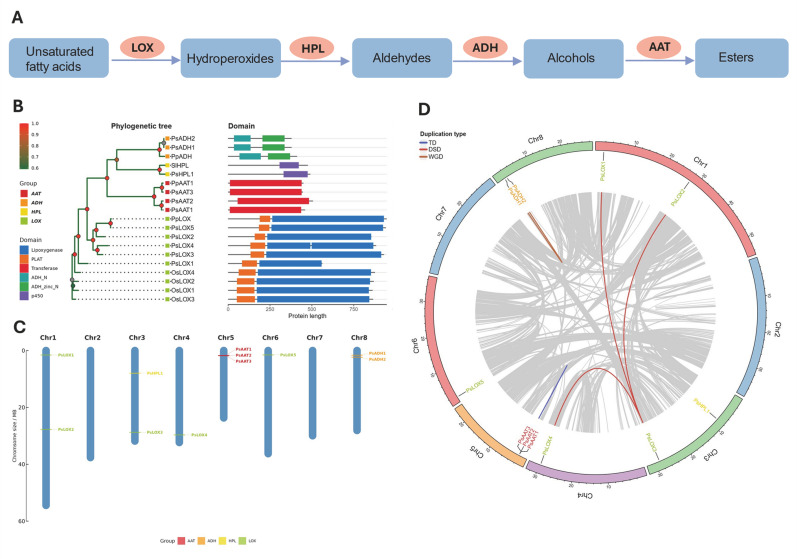
Identification and genomic characterization of candidate genes involved in fatty acid-derived ester biosynthesis (lipoxygenase–hydroperoxide lyase–alcohol dehydrogenase–alcohol acyltransferase; LOX-HPL-ADH-AAT). (**A**) Schematic overview of the fatty acid-derived LOX-HPL-ADH-AAT pathway leading to ester formation. (**B**) Phylogenetic relationships and conserved domain architectures of candidate *Prunus salicina* lipoxygenase (PsLOX), PsHPL, PsADH, and PsAAT proteins. Gene families are indicated by colors: LOX (green), HPL (yellow), ADH (orange), and AAT (red). The right panel shows protein domain architectures and protein length (aa, amino acids), with colored blocks representing different domain types. (**C**) Chromosomal (Chr) distribution of candidate genes in *Prunus salicina*. Blue bars represent chromosomes; gene positions are marked and colored as in (**A**). (**D**) Duplication analysis of candidate genes. Gray lines indicate genome-wide syntenic blocks; colored lines highlight syntenic gene pairs involving LOX/HPL/ADH/AAT candidates. Different colors correspond to duplication types (DSD, dispersed duplication; TD, tandem duplication; WGD, whole-genome duplication), as shown in the legend.

Using OrthoFinder-derived orthogroups, we identified orthologous candidates of the LOX-HPL-ADH-AAT pathway in Chinese plum. The pathway converts unsaturated fatty acids into volatile esters via LOX, HPL, ADH, and AAT (Figure 3A) [14]. Accordingly, we retrieved five *PsLOX*, one *PsHPL*, two *PsADH*, and three *PsAAT* genes as candidate members of this pathway (Figure 3B). Chromosomal mapping showed that these genes are distributed across multiple chromosomes, except Chr2 and Chr7: *PsLOX* members were located mainly on Chr1, Chr3, Chr4, and Chr6; *PsHPL* was located on Chr3; and *PsADH* genes were located on Chr8 (Figure 3C). Interestingly, *PsAAT1/2/3* formed a tight cluster on Chr5, suggesting a local duplication event. To clarify the evolutionary basis of this expansion, we examined duplication patterns and synteny relationships (Figure 3D). Three dispersed-duplication (DSD) gene pairs were detected within the *PsLOX* family, two whole-genome duplication (WGD) gene pairs within *PsADH*, and two tandem-duplication (TD) gene pairs within *PsAAT*.

### 2.4. Transcriptomic Comparison Between High-Ester ‘WW’ and Low-Ester ‘KLD’ Across Ripening Stages

**Figure 4:**
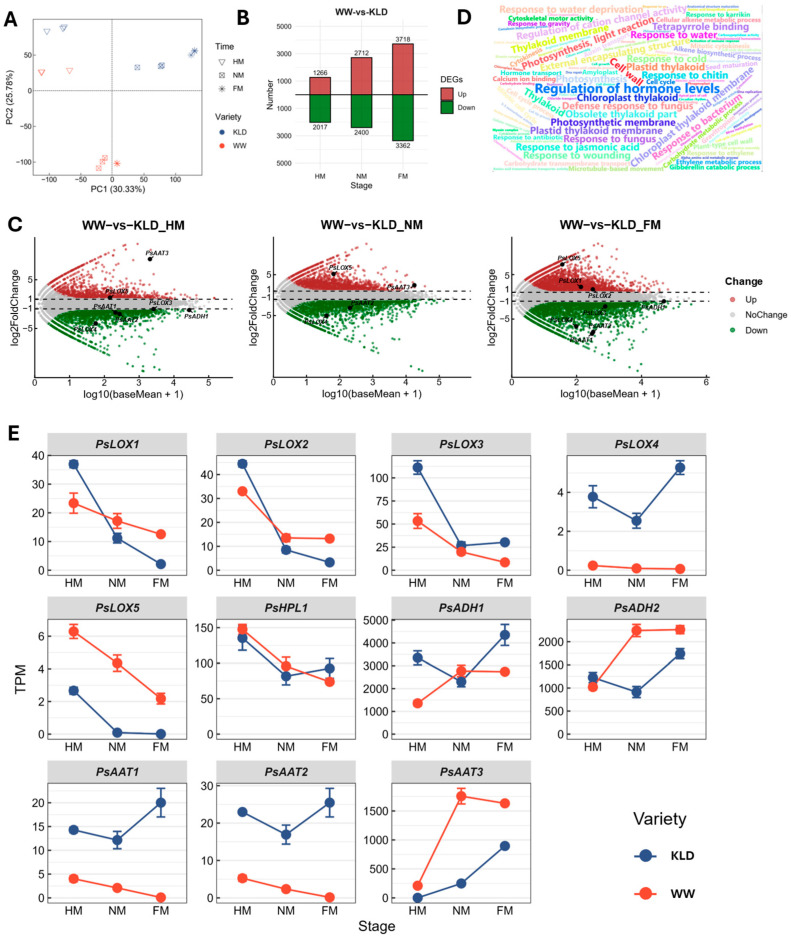
Transcriptomic comparison between the high-ester cultivar ‘WeiWang’ (WW) and the low-ester cultivar ‘KongLongDan’ (KLD) across three ripening stages. (**A**) Principal component analysis (PCA) of pulp transcriptomes at the half-mature (HM), near-mature (NM), and full-mature (FM) stages in ‘WW’ and ‘KLD’. (**B**) Numbers of differentially expressed genes (DEGs) between ‘WW’ and ‘KLD’ at each ripening stage. (**C**) Volcano plots of ‘WW’ vs. ‘KLD’ at HM, NM, and FM, highlighting key candidate genes associated with fatty acid-derived volatile biosynthesis. (**D**) Word cloud of Gene Ontology (GO) enrichment terms for DEGs; font size reflects enrichment significance and/or representativeness. (**E**) Expression levels of candidate genes in the lipoxygenase–hydroperoxide lyase–alcohol dehydrogenase–alcohol acyltransferase (LOX-HPL-ADH-AAT) pathway across stages in ‘WW’ and ‘KLD’. Data are presented as mean ± SD (*n* = 3).

To elucidate transcriptional differences between the high-ester cultivar ‘WW’ and the low-ester cultivar ‘KLD’ during ripening, and to relate these differences to fatty acid-derived volatile biosynthesis, we performed RNA-seq on pulp tissues collected at three ripening stages (HM, NM, and FM). Principal component analysis (PCA) showed that PC1 and PC2 explained 30.33% and 25.78% of the total variance, respectively. Biological replicates clustered tightly within each cultivar and stage, supporting the robustness of the dataset. HM samples were clearly separated from NM/FM samples, and ‘WW’ and ‘KLD’ were also separated within the same stage, indicating that both ripening progression and cultivar genotype contribute substantially to transcriptional divergence (Figure 4A).

Differentially expressed genes (DEGs) between ‘WW’ and ‘KLD’ were identified at each stage (|log2 fold change| ≥ 1, padj < 0.05), revealing an increasing number of DEGs as ripening progressed (Figure 4B,C and Table S2). Gene Ontology (GO) enrichment analysis indicated strong stage specificity in the biological processes distinguishing the two cultivars (Figure 4D). At the NM and FM stages, enriched GO terms were increasingly related to alcohol-associated processes (e.g., response to alcohol and cellular response to alcohol), isoprenoid metabolism, and response to jasmonic acid (Table S3).

Further analysis of the expression patterns of key genes in the LOX pathway revealed that, except for *PsHPL1*, which showed no significant difference between the two cultivars at any stage, the other genes generally exhibited stage-dependent, cultivar-specific differences (Figure 4C,E). Within the LOX family, *PsLOX1*, *PsLOX2*, *PsLOX3*, and *PsLOX5* generally decreased with ripening in both cultivars, but with distinct cultivar-specific patterns. Specifically, *PsLOX3* was downregulated in ‘WW’ relative to ‘KLD’ at HM and FM. *PsLOX1* and *PsLOX2* were lower in ‘WW’ at HM but became significantly higher than ‘KLD’ at NM and FM. *PsLOX4* showed overall low expression in ‘WW’ and was consistently downregulated across stages. In contrast, *PsLOX5* was significantly upregulated in ‘WW’ compared with ‘KLD’ at all three stages.

For ADH genes, *PsADH1* was significantly lower in ‘WW’ than in ‘KLD’ at HM and FM, whereas no significant difference was detected at NM. *PsADH2* peaked at NM in ‘WW’ and remained relatively high at FM, and its expression was significantly higher in ‘WW’ than in ‘KLD’ at both NM and FM. For AAT genes, *PsAAT1* and *PsAAT2* decreased progressively with ripening and were overall downregulated in ‘WW’ relative to ‘KLD’, with the largest difference at FM. In particular, *PsAAT3* exhibited substantially higher transcript abundance in pulp than *PsAAT1*/2. In ‘WW’, *PsAAT3* increased sharply from HM to NM and remained high at FM; it was consistently upregulated in ‘WW’ relative to ‘KLD’, with the strongest difference at NM (Figure 4E). In summary, the expression pattern of PsAAT3 showed a clear association with ester accumulation between the high-ester cultivar ‘WW’ and the low-ester cultivar ‘KLD’. In addition, AAT catalyzes the final step of ester biosynthesis. Taken together, these results suggest that PsAAT3 is a strong candidate gene associated with ester differences among cultivars and was therefore selected for transient overexpression analysis. In addition, Quantitative Real-Time PCR (qRT-PCR) validation of *PsAAT1*, *PsAAT2*, *PsAAT3*, *PsADH2*, *PsLOX4*, and *PsLOX5* showed expression trends consistent with RNA-seq results (Figure S2), supporting the reliability of the transcriptome results.

### 2.5. Transient Overexpression of PsAAT3 Promotes Ester Volatile Production

**Figure 5:**
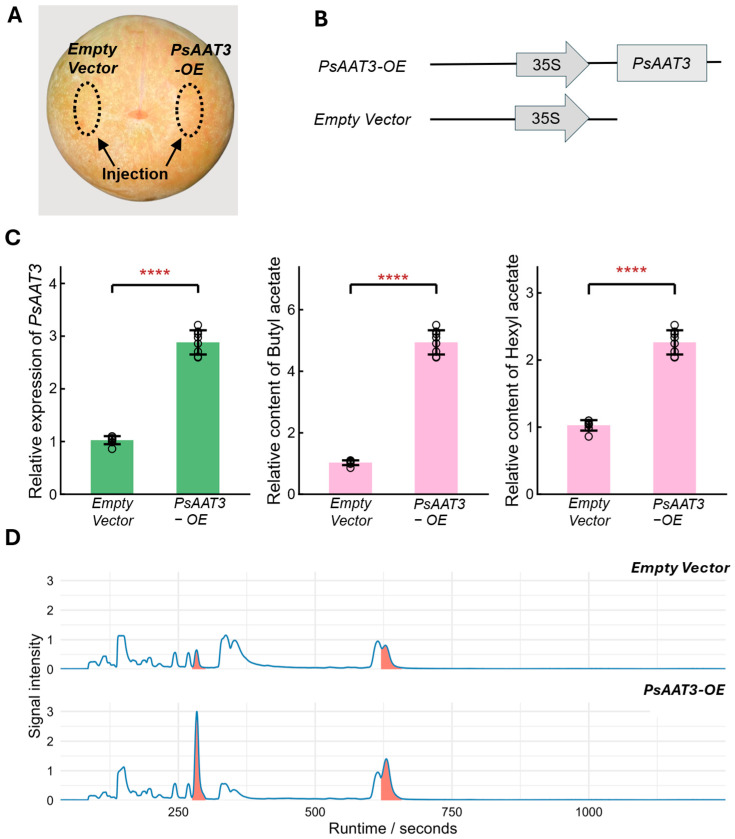
Transient overexpression of *Prunus salicina* alcohol acyltransferase 3 (*PsAAT3*) promotes ester volatile accumulation in plum fruit. (**A**) Schematic of the transient overexpression infiltration assay: the empty vector control and *PsAAT3* overexpression construct (*PsAAT3-OE*) were injected into opposite sides of the same fruit (dashed circles indicate infiltration sites). (**B**) Vector map of the cauliflower mosaic virus 35S promoter (CaMV 35S)-driven *PsAAT3* overexpression construct and the empty vector control. (**C**) Quantitative real-time PCR (qRT-PCR) validation of *PsAAT3* overexpression and gas chromatography–mass spectrometry (GC-MS)-based semi-quantitative analysis of ester volatiles in infiltrated tissues. Data are presented as mean ± SD (standard deviation, *n* = 3 biological replicates). Asterisks indicate significant differences between *PsAAT3*-OE and empty vector (****, *p* < 0.0001). Comparisons were conducted using two-tailed Student’s *t*-tests. (**D**) Representative chromatograms from pulp samples infiltrated with *PsAAT3*-OE and the empty vector.

To examine *PsAAT3* involvement in ester biosynthesis, we assembled an overexpression plasmid by cloning *PsAAT3* downstream of the CaMV 35S promoter in the pGreenII 62-SK vector; the empty vector served as the negative control (Figure 5B). Low-ester cultivar ‘KLD’ was used as materials, Agrobacterium suspension carrying the empty vector and the *PsAAT3* overexpression construct were injected on the opposite side of the same fruit (Figure 5A). Five days after injection, pulp tissue from the injected regions was collected. qRT-PCR confirmed that *PsAAT3* transcript abundance was significantly elevated in the overexpression treatment, reaching ~2.8-fold of the empty vector control (Figure 5C). Consistently, GC-MS profiling showed markedly enhanced chromatographic signals for butyl acetate and hexyl acetate in the overexpression samples (Figure 5D). Their abundances increased to 4.8-fold and 2.2-fold of the control, respectively (Figure 5C), supporting a positive role of *PsAAT3* in ester biosynthesis at the metabolic level. Overall, transient overexpression of *PsAAT3* substantially increased ester levels in a low-ester genetic background, providing direct functional evidence that *PsAAT3* is a key determinant contributing to ester divergence between high- and low-ester cultivars.

### 2.6. WGCNA Reveals Ester-Associated Modules and Putative Regulatory Factors

**Figure 6:**
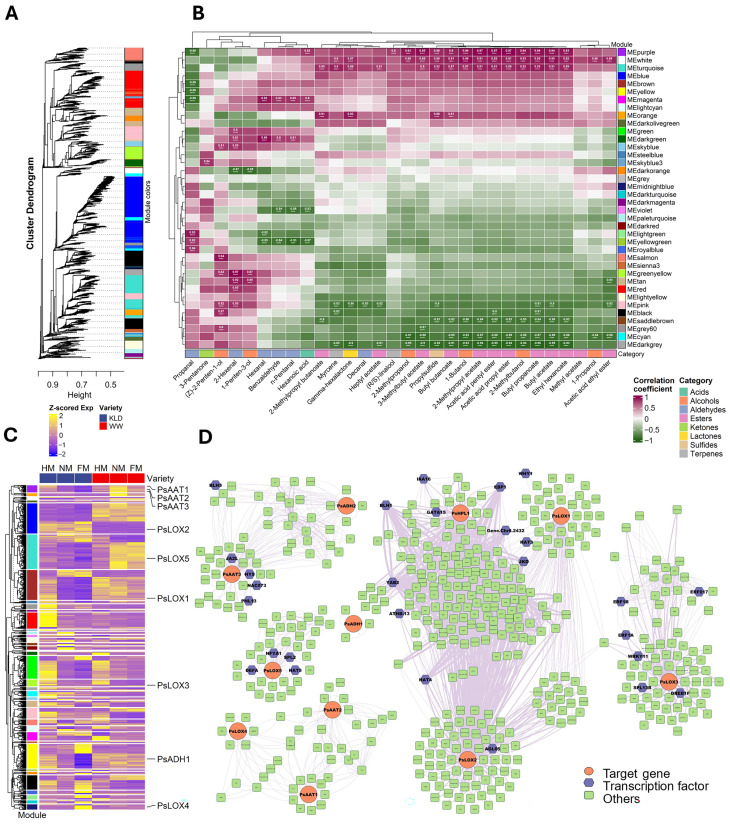
Weighted gene co-expression network analysis (WGCNA) and co-expression network analysis revealing regulatory networks associated with volatile accumulation in ‘WeiWang’ (WW) and ‘KongLongDan’ (KLD). (**A**) Hierarchical clustering dendrogram of differentially expressed genes (DEGs), with 39 co-expression modules indicated by color bars. (**B**) Module-trait relationship heatmap showing correlations between module eigengenes (MEs) and volatile organic compounds (VOCs) relative abundances. The heatmap reports *r* values for each module-trait pair, and statistical significance is indicated by asterisks: * *p* < 0.05, ** *p* < 0.01, *** *p* < 0.001, and **** *p* < 0.0001. (**C**) Heatmap of module expression profiles (z-score normalized), with the module assignments of representative fatty acid-derived pathway genes indicated on the right. (**D**) Co-expression network centered on key genes in the lipoxygenase–hydroperoxide lyase–alcohol dehydrogenase–alcohol acyltransferase (LOX-HPL-ADH-AAT) pathway. Orange nodes indicate pathway genes, blue nodes indicate transcription factors, and green nodes indicate other co-expressed genes; edge thickness reflects co-expression strength. Abbreviations: r, correlation coefficient; z-score, standard score.

To better associate transcriptional divergence between ‘WW’ and ‘KLD’ with VOC accumulation, WGCNA was applied by integrating 8840 DEGs with 30 VOC traits, which yielded 39 co-expression modules (Figure 6A). Module sizes were highly uneven: turquoise was the most gene-rich group (1066 members), while gray comprised only two genes (Table S4). Correlating module eigengenes with metabolite traits showed that the purple, white, and turquoise modules were consistently and positively associated with the majority of ester volatiles (*p* < 0.05), with correlation values spanning 0.80–0.97 (Figure 6B). In particular, *PsAAT1*, *PsAAT2*, and *PsAAT3* were assigned to the purple module, while *PsLOX5* was assigned to the turquoise module (Figure 6C). In contrast, the cyan, saddlebrown, and darkgrey modules were significantly negatively correlated with most esters (*p* < 0.05), with correlation coefficients ranging from −0.80 to −0.96 (Figure 6B). We then constructed a co-expression regulatory network centered on key genes in the fatty acid-derived pathway (Figure 6D). Several pathway genes showed strong co-expression connections with TFs. For example, candidate transcription factors co-expressed with *PsAAT3* included *JA2L*, *HY5*, *NAC073*, and *PHL13*, which were further supported by the predicted transcription factor binding sites identified in the *PsAAT3* promoter analysis (Figure S4). The candidate transcription factors co-expressed with *PsLOX5* included *DEFA*, *NFYA1*, *SPL2*, and *HAT5*.

## 3. Discussion

### 3.1. Ripening and Cultivar Effects on VOCs and Aroma

Fruit aroma is a key sensory attribute influencing overall flavor quality and consumer preference, and it is largely determined by the composition and relative abundance of VOCs [5]. In this study, VOC profiling of pulp tissues from three plum cultivars showed that esters, aldehydes, and alcohols were the predominant chemical classes, suggesting that fatty acid-derived volatile contribute substantially. This pattern agrees with previous studies in plum, which also reported esters, aldehydes, and alcohols as the major VOC classes [29]. In the pulp of all three cultivars, hexyl acetate, butyl acetate, and ethyl hexanoate were the most abundant esters. Many studies have also described these three esters as key “fruity-note” compounds in different fruit species [29,30,31].

Previous studies show that immature fruits often contain more aldehydes and alcohols that give green or grassy notes. As fruits ripen, aldehydes and alcohols usually decrease, while esters build up. This shift often changes the aroma from green to fruity and sweet [31,32,33]. For example, it has been reported in peach and pear, where ripening-related decreases in alcohol and marked increases in esters enhance overall fruitiness [17,34]. Consistent with this pattern, the two ester-dominant cultivars ‘WW’ and ‘WD’ showed a clear increase in ester abundance during ripening and exhibited a fruity-dominant sensory profile (Figure S1), suggesting that esters constitute the principal aroma “backbone” in these cultivars.

However, the patterns of flavor formation differed between cultivars: ‘WD’ showed its most prominent sensory features mainly at the FM stage, with herbal/banana-like notes becoming more evident, whereas ‘WW’ displayed a broader range of dominant flavors and a more pronounced transition from green-associated notes at HM to a complex fruity profile at later stages, implying a stronger capacity for aroma release during late ripening. Many studies in other fruit species have reported a similar pattern, where differences in VOC abundance between cultivars lead to clear differences in sensory aroma traits [34,35,36]. In contrast, ‘KLD’ showed only small changes in the proportion of alcohols, aldehydes, and esters, and it did not show a clear trend across ripening. In ‘KLD’, green-note and fruity-note volatiles contributed at similar levels, and the fruit did not show a typical shift from “green” to “fruity” aroma. Studies in fig and grape have reported similar patterns [37,38]. Taken together, ‘KLD’ shows only a weak ripening-associated rise in ester accumulation, likely due to limited synthesis and/or faster turnover. This pattern may explain the modest increase in fruitiness and the limited reduction in green-related volatiles.

### 3.2. The LOX Pathway Shapes Ester Accumulation

In fruit, many aldehydes, alcohols, and volatiles come from the LOX pathway of fatty acid metabolism [39]. In this pathway, LOX and HPL generate C6/C9 aldehyde precursors. ADH then converts aldehydes and alcohols and helps supply alcohol substrates. AAT transfers an acyl group from acyl-CoA to an alcohol and produces volatile esters [14]. In our study, homology-based mining of LOX-pathway genes showed that *PsAAT1*, *PsAAT2*, and *PsAAT3* form a tandem cluster on the chromosome. This pattern suggests that local gene duplication may have expanded and diversified esterification capacity in Chinese plum. Several fruit species show a similar link between tandem AAT loci and ester variation. In peach, sequence variation within a tandem AAT region is associated with the loss of (Z)-3-hexenyl acetate production [20]. In kiwifruit, the HiFI locus, which contains tandem AAT genes, has been shown to drive the formation of multiple volatile esters [40]. In apple, allelic variation at the *AAT1* locus results in marked segregation of ester profiles in hybrid populations, and *AAT1* downregulation in high-ester cultivars causes strong reductions in multiple key esters [41]. Together, these genetic and functional studies support AAT as a central node controlling ester accumulation.

Importantly, AAT family members often exhibit distinct regulation patterns during ripening, reflecting divergence in regulation and/or substrate preference. For example, in apricot and peach, *PaAAT1* and *PpAAT1* (orthologous to *PsAAT3* in this study in Figure 3B) show higher and/or ripening-specific expression [16,20]. In agreement, our transcriptomic data showed that *PsAAT3* displayed substantially higher expression than *PsAAT1*/2, and it was strongly induced during ripening in the high-ester cultivar ‘WW’, whereas *PsAAT1*/2 decreased with ripening and were overall lower in ‘WW’. Furthermore, transient overexpression of PaAAT1 in apricot promotes the accumulation of several key esters (e.g., hexyl acetate), although its effect can vary among individual esters. In our study, transient overexpression of *PsAAT3* in a low-ester background significantly increased hexyl acetate and butyl acetate, providing direct functional evidence that *PsAAT3* is a key factor explaining cultivar differences in ester accumulation.

Precursor supply also affects ester production. The LOX-HPL-ADH module sets the aldehyde and alcohol pools that feed the AAT reaction. Many studies on fruits report that changes in key upstream genes are often associated with cultivar differences in ester levels and with ripening-related changes in esters. In “Nanguo” pear during postharvest ripening, the expression of *PuLOX3*, *PuADH3*, and *PuAAT* shows strong positive correlations with key ester volatiles [39]. In apple callus, *MdLOX1a* overexpression increases volatile ester levels [15]. In passion fruit, studies also suggest that the increased transcriptional levels of specific LOX members during ripening may relate to ester accumulation [42]. In addition, Li [43] reported that *PuFAD2*, *PuLOX2*, *PuLOX5*, and *PuAAT* are closely linked to high ester accumulation in “Nanguo” pear. We found a comparable association in our study. *PsLOX4*, *PsLOX5*, and *PsADH2* were differentially expressed in the high-ester cultivar ‘WW’. WGCNA further assigned *PsLOX5* to the turquoise module, which showed strong positive correlations with most ester abundances. Together, these results indicate that upstream LOX-pathway activity may contribute to ester accumulation in plum.

### 3.3. Transcription Factors Regulating Ester Biosynthesis

In recent years, many studies have identified transcription factors (TFs) that regulate VOC biosynthesis in fruit. Our co-expression network (Figure 6D) shows that key genes in the fatty acid-derived LOX pathway are closely co-expressed with multiple TF families. Many studies also suggest that transcriptional control of AAT genes is important for the abundance of fruity esters. In our dataset, *PsAAT3* shows strong co-expression with *NAC073*, which suggests a regulatory connection. Molecular evidence supports NAC-mediated activation of AAT: in peach, *PpNAC1* directly binds the NACBS element in the *PpAAT1* promoter and enhances transcription, and its overexpression significantly increases ester accumulation [44]. NAC regulators have also been implicated in AAT regulation in apple (e.g., *MdNAC5*-*MdAAT1*). Together, these observations suggest that *NAC073* may contribute to *PsAAT3* transcriptional activation, thereby enhancing terminal esterification and ester accumulation in plum.

In addition, co-expression between *JA2L* and *PsAAT3* suggests potential involvement of jasmonate signaling in regulating terminal esterification. In apple, exogenous JA induces *MdMYC2* and coordinately activates multiple LOX-pathway genes [31], supporting a link between JA-related transcriptional networks and volatile formation. Co-expression between *HY5* and *PsAAT3* further implies a possible contribution of light-responsive regulation to AAT activation. Moreover, diverse TF families-including bZIP, WRKY, and MADS-have been reported to regulate AAT via promoter binding in pear, banana, and peach, and to display expression changes consistent with ester accumulation under postharvest ripening or low-temperature conditions [45,46,47]. However, the regulatory relationships between these TFs and PsAAT3 in plum still require direct experimental validation.

Ester accumulation is also constrained by aldehyde/alcohol precursor supply. In our network, *PsLOX5* showed co-expression with candidate factors such as *NFYA1*, *SPL2*, *HAT5*, and *DEFA*. Although their direct roles in aroma regulation remain to be validated, cross-species evidence indicates that upstream nodes such as LOX and ADH can be regulated by TFs (e.g., WRKY, ABI5) [48], thereby influencing volatile formation. In tomato, the ripening regulator RIN (a MADS-box TF) regulates LOX/HPL/ADH and other volatile-related genes [49], and in peach, ERF complexes enhance aroma formation by upregulating *LOX4*, suggesting that ethylene-associated transcriptional networks can simultaneously influence precursor supply and esterification potential [8]. Collectively, these findings support a model in which cultivar-specific ester accumulation arises from coordinated regulation of upstream precursor formation and *PsAAT3*-mediated terminal esterification.

## 4. Materials and Methods

### 4.1. Plant Materials

Fruits were harvested from the Jiamu National Long-Term Scientific Research Base for Pomology in Xinjiang. Three aromatic Chinese plum (*Prunus salicina*) cultivars, ‘WD’ (‘Weidi’), ‘WW’ (‘Weiwang’), and ‘KLD’ (‘Konglongdan’), grafted onto *Prunus davidiana* (Carrière) Franch. rootstock, were used in this study. For each cultivar, three healthy 7-year-old fruit-bearing trees with uniform vigor and no visible pests or diseases were selected as biological replicates (*n* = 3). Fruits were hand-harvested at three ripening stages: half-mature (HM), near-mature (NM), and full-mature (FM). Fruit developmental stages were determined according to days after full bloom (DAFB) and further characterized by quantitative measurements of soluble solids content (SSC), firmness, and fruit acidity at each sampling stage (Table S9). At each stage, 10 fruits were hand-collected from different canopy positions of each tree to constitute one biological replicate. Only fruits with uniform size and without insect damage or mechanical injury and consistent maturity were selected. After harvest, pulp tissues were separated, snap-frozen in liquid nitrogen, and stored at −80 °C for VOC analysis and transcriptome sequencing.

### 4.2. Volatile Organic Compound (VOC) Analysis

Plum pulp volatiles were analyzed using headspace solid-phase microextraction coupled to gas chromatography-mass spectrometry (HS-SPME-GC-MS). A 0.2 g pulp tissue was ground in liquid nitrogen and immediately transferred into a 20 mL headspace vial. The sealed vial was equilibrated at 50 °C for 20 min. VOCs in the headspace were trapped with a conditioned PDMS/DVB SPME fiber (65 μm; Supelco, Bellefonte, PA, USA), which was then thermally desorbed in the GC-MS injection port to acquire chromatographic and mass spectral signals. Separation was performed on a DB-5 capillary column (30 m × 0.25 mm × 0.25 μm) with helium (≥99.999%) as the carrier gas at 1.0 mL·min−1. The oven temperature program started at 40 °C (3.5 min), increased to 250 °C at 10 °C·min^−1^, and was held at the final temperature as described previously. The mass spectrometer operated in EI mode (70 eV) and collected full-scan spectra over m/z 50–500; the ion source, quadrupole, and transfer line were set to 230 °C, 150 °C, and 280 °C, respectively [25,50]. For compound identification, a C7-C40 n-alkane series was run under the same GC conditions to calculate retention indices (RI). Mass spectra were searched against the National Institute of Standards and Technology (NIST) mass spectral library [51], and hits with similarity scores > 80% were accepted. Peak detection and area integration were performed in Xcalibur 2.0. Because no internal standard was added, VOCs were reported as semi-quantitative relative abundances based on peak areas, and the peak areas were normalized across samples to reduce variation introduced during sampling and injection. The relative abundance of each VOC class was calculated by summing the normalized abundances of compounds within that class. Heatmaps were generated from the normalized relative abundance matrix. Each compound was scaled across samples using a row-wise z-score for visualization. Hierarchical clustering was carried out in R (v4.5.1) using pheatmap (v1.0.13) with Euclidean distance and Ward.D2 linkage, which was used to compare sample profiles and highlight changes in key volatiles.

### 4.3. Sensory Attribute Profiling Based on VOC Annotations

To characterize overall flavor attributes, volatile compounds were categorized into flavor descriptors in The Good Scents Company database (https://www.thegoodscentscompany.com/) (Table S6). For each sample, normalized relative abundances of compounds within the same sensory dimension were summed to obtain the within-sample intensity for that dimension. The summed values were then normalized across all samples to generate relative intensity scores for comparisons among cultivars and ripening stages. Aroma radar plots were generated using ggplot2 (v4.0.0) in R.

### 4.4. Identification of Orthologous Genes in the Fatty Acid-Derived Volatile Pathway

Reference protein sequences for key enzymes in the fatty acid-derived volatile pathway were retrieved from UniProt. ADH and AAT reference sequences were obtained from peach (*Prunus persica*), while the HPL reference sequence was obtained from tomato (*Solanum lycopersicum*). To improve coverage and representativeness of LOX homolog searches, LOX reference sequences were mainly from peach and supplemented with well-annotated, functionally characterized LOX proteins from rice (*Oryza sativa*). UniProt accessions, species sources, and annotations of all reference proteins are listed in Table S7.

The plum reference genome and protein annotation files were obtained from the *Prunus salicina* ‘Zhongli No. 6′ Genome v1.0 assembly (https://www.rosaceae.org/Analysis/9019655, accessed on 15 October 2024). The *P. salicina* proteome, together with the proteomes of reference species were used as input for cross-species orthology inference using OrthoFinder (v2.5.5) to identify orthogroups. Based on the orthogroups to which the reference proteins were assigned, the corresponding *P. salicina* members were retrieved as a candidate sequence set. Candidate proteins were then annotated for conserved domains using PfamScan (pfam_scan.pl v1.6; Pfam-A), and sequences containing the characteristic Pfam domains of the target family were retained as the final members. Multiple sequence alignment was performed using MAFFT (v7.526), and an approximately maximum-likelihood phylogenetic tree was constructed using FastTree (v2.2.0). Phylogenies were visualized in R using ggtree. Chromosomal locations of target genes were plotted with TBtools (v2.0). Duplication types were inferred and classified using the TBtools Duplicate Gene Pair Finder module in combination with genome collinearity information, and results were visualized accordingly.

### 4.5. RNA-Seq and Differential Expression Analysis

Total RNA was isolated from pulp samples with TRIzol (Invitrogen, Carlsbad, CA, USA). An Agilent 2100 Bioanalyzer (Agilent Technologies, Santa Clara, CA, USA) was used to assess RNA integrity and quality. RNA-seq libraries were constructed according to the kit instructions. Sequencing was performed by BGI (Shenzhen, China) on an Illumina HiSeq X Ten platform to generate 150 bp paired-end reads. The pipeline removed adapter reads and excluded low-quality sequences. The retained clean reads were mapped to the *P. salicina* reference genome (Zhongli No. 6 v1.0; the same assembly described in Section 4.4) with HISAT2 (v2.2.1). FeatureCounts was applied to summarize reads at the gene level and generate the raw count table. DESeq2 (v1.50.2) was used for differential expression testing based on raw counts. The Benjamini–Hochberg procedure was applied for multiple-testing correction. Genes were defined as DEGs when |log2 fold change| ≥ 1 and padj < 0.05. TPM values were calculated only for expression visualization. PCA was performed in R based on variance-stabilized values from DESeq2. Functional annotation was carried out with eggNOG (v5.0). GO enrichment was tested using Fisher’s exact test with Benjamini–Hochberg correction (padj < 0.05).

### 4.6. Quantitative Real-Time PCR (qRT-PCR)

qRT-PCR was carried out to confirm the RNA-seq results. Total RNA was isolated with the EZ-10 RNA extraction kit (Sangon Biotech, Shanghai, China). RNA concentration and A260/A280 ratios were assessed using a NanoDrop 2000 spectrophotometer (Thermo Fisher Scientific, Waltham, MA, USA). A 1% agarose gel was used to check RNA integrity. First-strand cDNA was synthesized from 800 ng total RNA using the ReverTra Ace qPCR RT Kit (TOYOBO, Osaka, Japan) under the supplier’s conditions (37 °C for 15 min, followed by 98 °C for 5 min). ACT2 was selected as the reference gene [26]. Primer Premier 5 was used to design primers for qRT-PCR (Table S8). Reactions were run with SYBR Premix Ex Taq II (TaKaRa, Dalian, China) on a LightCycler 480 II instrument (Roche Diagnostics, Basel, Switzerland). Relative transcript levels were calculated with the 2^−ΔΔCt^ method. Each treatment included three biological replicates, and each replicate was measured with four technical repeats.

### 4.7. WGCNA and Co-Expression Network Construction

Weighted gene co-expression network analysis (WGCNA) was conducted in R using WGCNA (v1.73) with 8840 DEGs and the relative abundances of 30 VOCs. The soft-thresholding power was chosen using the scale-free topology criterion (power = 11; R2 > 0.85). An unsigned network was constructed using bicor (biweight midcorrelation). The adjacency matrix was converted into a topological overlap matrix (TOM). Genes were hierarchically clustered using TOM dissimilarity, modules were detected by dynamic tree cutting, and similar modules were merged according to module eigengene (ME) similarity. Module-trait associations were assessed by correlating MEs with VOC traits using bicor and visualized as a heatmap for all modules; modules of interest were further prioritized using |cor| ≥ 0.8 and *p* ≤ 0.05 for downstream analyses. Hub genes were identified based on high module membership (kME). For network visualization, edges were filtered by retaining the top 5–10% TOM/adjacency weights, and networks were plotted in R (v4.5.1).

### 4.8. Construction of the PsAAT3 Overexpression Vector and Transient Expression Assay

Primers targeting the full-length *PsAAT3* coding sequence were designed in SnapGene according to the SpeI/EcoRI cloning sites in pGreenII 62-SK (Forward: GGCGGCCGCTCTAGAACTAGTATGGGTTCATTGTGCCCTCTAC; Reverse: GATAAGCTTGATATCGAATTCTTACTCCTCGACATGCTCCAAT). The *PsAAT3* CDS was amplified from pulp cDNA of ‘KLD’. The pGreenII 62-SK backbone was digested with SpeI and EcoRI, followed by gel purification. The *PsAAT3* fragment was then ligated into the vector using the ClonExpress II One Step Cloning Kit (Vazyme, Nanjing, China) to obtain a CaMV 35S-driven overexpression plasmid (*PsAAT3-OE*), while the empty vector was used as the negative control. The overexpression constructs were transformed into Agrobacterium tumefaciens GV3101. Transformed GV3101 cells were collected, resuspended in liquid MS medium, and adjusted to an OD600 of ~0.8. The cultivar ‘KLD’ was used for transient expression because it shows a low-ester phenotype and has a well-established fruit infiltration system. Ten ‘KLD’ fruits at the HM stage, free of mechanical damage and of uniform size, were selected. On opposite sides of each fruit, a sterile 1 mL syringe was used to puncture the peel, and the needle was inserted approximately 3–4 mm beneath the peel into the fruit tissue. One milliliter of the *PsAAT3-OE* or empty vector bacterial suspension was slowly injected into each side. The experiment was repeated three times [52]. The infiltrated fruits were maintained at 25 °C. After 5 days, pulp tissues from the labeled regions were excised, snap-frozen in liquid nitrogen, and stored at −80 °C. Half of the sample was used for RNA extraction and qRT-PCR to validate the overexpression of *PsAAT3*. The remaining tissues were used for VOC analysis.

### 4.9. Statistical Analysis and Visualization

Except for the transcriptome and co-expression network analyses, all statistical processing and data visualization were carried out in R (v4.5.1). The study used a two-tailed Student’s *t*-test for two-group comparisons and one-way ANOVA with Tukey’s HSD for post hoc tests when three or more groups were compared. The significance threshold was set at *p* < 0.05.

## 5. Conclusions

This study systematically characterized ripening-associated changes in VOC profiles and their molecular basis in three Chinese plum (*Prunus salicina*) cultivars. We identified 38 VOCs, and esters, aldehydes, and alcohols were the main classes. The high-ester cultivar ‘WW’ showed higher total VOC abundance and much stronger ester accumulation than ‘WD’ and ‘KLD’. In pulp, hexyl acetate, butyl acetate, and ethyl hexanoate were the most abundant key esters. These changes were accompanied by a ripening-related flavor shift, with weaker green notes and stronger fruity aroma. Gene expression analysis of the main pathway showed clear cultivar- and stage-dependent differences for most LOX-ADH-AAT genes, but *PsHPL1* did not differ between cultivars. *PsAAT3* showed the highest transcript level in pulp and was strongly induced during ripening in ‘WW’. These results identify *PsAAT3* as a core candidate gene that underlies cultivar differences in ester accumulation. Transient overexpression of *PsAAT3* significantly increased butyl acetate and hexyl acetate in the low-ester cultivar ‘KLD’, which provides direct evidence that *PsAAT3* promotes ester formation. WGCNA further identified purple and turquoise modules that were strongly associated with ester traits and highlighted co-expressed transcription factors, offering leads for future work on transcriptional control of ester aroma formation.

## Data Availability

The RNA-seq data produced and used for this work are publicly accessible via the NCBI Sequence Read Archive (SRA) with the project accession PRJNA1424178. All remaining datasets that underpin the conclusions are provided within the manuscript and its Supplementary Materials. Further details can be obtained from the corresponding authors upon a justified request.

## Supplementary Materials

The following supporting information can be downloaded at: https://www.mdpi.com/article/10.3390/plants15081144/s1, Figure S1. Aroma descriptor profiles across ripening stages shown separately for each cultivar. Each axis represents one aroma category, and the radius indicates its relative intensity. For each cultivar, intensities were normalized within that cultivar, and the outer circle represents the highest aroma intensity observed across all stages and all aroma categories in that cultivar. All values are scaled relative to this cultivar-specific maximum. Colored lines represent different ripening stages; Figure S2. Quantitative Real-Time PCR (qRT-PCR) validation of expression levels for selected ester-related differentially expressed genes (DEGs); Figure S3. Statistical comparison of major volatile organic compound (VOC) classes across ripening stages in three plum cultivars. Relative contents of acids, alcohols, aldehydes, esters, ketones, lactones, sulfides, and terpenes are shown for ‘KongLongDan (KLD)’, ‘WeiDi (WD)’, and ‘WeiWang (WW)’ at the half-mature (HM), near-mature (NM), and full-mature (FM) stages. Data are presented as mean ± SD. Different letters indicate significant differences among samples at *p* < 0.05; Figure S4. Distribution of predicted cis-elements associated with candidate transcription factor families in the 2000 bp promoter region of *Prunus salicina* alcohol acyltransferase 3 (PsAAT3). Predicted NAC-, MYB-related, ERF-, and bZIP-related cis-elements are shown according to their positions upstream of the transcription start site (TSS); Table S1: Normalized peak areas of VOCs in ‘WD’, ‘WW’, and ‘KLD’ across maturity stages; Table S2: Differentially expressed genes (DEGs) between ‘WW’and ‘KLD’across three maturity stages (HM, NM, and FM); Table S3: Gene Ontology (GO) enrichment results of DEGs used for functional interpretation and visualization; Table S4: Summary of WGCNA modules and key genes associated with aroma-related pathways; Table S5: Sampling time points of ‘WD’, ‘WW’, and ‘KLD’ at three maturity stages (HM, NM, and FM) expressed as days after full bloom (DAFB); Table S6: Flavor descriptors corresponding to identified VOCs; Table S7: Accession numbers and related information for reference protein sequences used in this study; Table S8: Primer sequences used for qRT-PCR analysis of key genes involved in the LOX-HPL-ADH-AAT pathway in *Prunus salicina*; Table S9: Tree age, and days after full bloom (DAFB) maturity indices of three Chinese plum cultivars at different ripening stages used for fruit sampling. The table includes tree age, days after full bloom (DAFB), soluble solids content (SSC), firmness, and fruit acidity, which were used to support stage classification and harvest criteria.

## References

[1] Bonany J., Buehler A., Carbó J., Codarin S., Donati F., Echeverria G., Egger S., Guerra W., Hilaire C., Höller I. (2013). Consumer Eating Quality Acceptance of New Apple Varieties in Different European Countries. Food Qual. Prefer..

[2] Klee H.J., Tieman D.M. (2018). The Genetics of Fruit Flavour Preferences. Nat. Rev. Genet..

[3] Pineli L.L.O., Moretti C.L., Dos Santos M.S., Campos A.B., Brasileiro A.V., Córdova A.C., Chiarello M.D. (2011). Antioxidants and Other Chemical and Physical Characteristics of Two Strawberry Cultivars at Different Ripeness Stages. J. Food Compos. Anal..

[4] Zhang B., Xi W., Wei W., Shen J., Ferguson I., Chen K. (2011). Changes in Aroma-Related Volatiles and Gene Expression during Low Temperature Storage and Subsequent Shelf-Life of Peach Fruit. Postharvest Biol. Technol..

[5] Lu H., Zhao H., Zhong T., Chen D., Wu Y., Xie Z. (2024). Molecular Regulatory Mechanisms Affecting Fruit Aroma. Foods.

[6] Li L., Fang Y., Li D., Zhu Z.H., Zhang Y., Tang Z., Li T., Chen X.S., Feng S.Q. (2023). Transcription Factors *MdMYC2* and *MdMYB85* Interact with Ester Aroma Synthesis Gene *MdAAT1* in Apple. Plant Physiol..

[7] Fang X., Shen J., Zhang L., Zou X., Jin L. (2024). Metabolomic and Transcriptomic Integration Reveals the Mechanism of Aroma Formation as Strawberries Naturally Turn Colors while Ripening. Food Chem..

[8] Wang Q., Gao F., Chen X., Wu W., Wang L., Shi J., Huang Y., Shen Y., Wu G., Guo J. (2022). Characterization of Key Aroma Compounds and Regulation Mechanism of Aroma Formation in Local Binzi (*Malus pumila* × *Malus asiatica*) Fruit. BMC Plant Biol..

[9] Rey-Serra P., Mnejja M., Monfort A. (2022). Inheritance of Esters and Other Volatile Compounds Responsible for the Fruity Aroma in Strawberry. Front. Plant Sci..

[10] Zhang Z., Yin Z. (2023). The Aroma Volatile in ‘Nanguo’ Pear: A Review. Horticulturae.

[11] Niu Y., Wang R., Xiao Z., Zhu J., Sun X., Wang P. (2019). Characterization of ester odorants of apple juice by gas chromatography-olfactometry, quantitative measurements, odour threshold, aroma intensity and electronic nose. Food Res. Int..

[12] Dudareva N., Klempien A., Muhlemann J.K., Kaplan I. (2013). Biosynthesis, Function and Metabolic Engineering of Plant Volatile Organic Compounds. New Phytol..

[13] Zhu X., Xu X., Jiang F., Li Q., Zhang A., Li J., Zhang H. (2024). Insights into the aroma volatiles and the changes of expression of ester biosynthesis candidate genes during postharvest storage of European pear. Front. Plant Sci..

[14] Zhang B., Shen J.Y., Wei W.W., Xi W.P., Xu C.J., Ferguson I., Chen K. (2010). Expression of Genes Associated with Aroma Formation Derived from the Fatty Acid Pathway during Peach Fruit Ripening. J. Agric. Food Chem..

[15] Zhang J., Wang Y., Zhang S., Zhang S., Liu W., Wang N., Fang H., Zhang Z., Chen X. (2024). ABIOTIC STRESS GENE 1 Mediates Aroma Volatiles Accumulation by Activating *MdLOX1a* in Apple. Hortic. Res..

[16] Zhou W., Kong W., Yang C., Feng R., Xi W. (2021). Alcohol Acyltransferase Is Involved in the Biosynthesis of C6 Esters in Apricot (*Prunus armeniaca* L.) Fruit. Front. Plant Sci..

[17] Zhou F., Wang X., Wang Y., Wang Z., Zhang H., Zhang C., Zhai R., Yang C., Wang Z., Xu L. (2025). The *PcbZIP44*-*PcWRKY70* Module Mediates Volatile Ester Biosynthesis via *PcAAT1* Transcriptional Regulation during Pear Postharvest Ripening. J. Agric. Food Chem..

[18] Zhang L., Zhou K., Wang M., Li R., Dai X., Liu Y., Jiang X., Xia T., Gao L. (2022). The Functional Characterization of Carboxylesterases Affecting the Catabolism of Volatile Esters in Strawberry. Int. J. Mol. Sci..

[19] Martínez-Rivas F.J., Blanco-Portales R., Moyano E., Alseekh S., Caballero J.L., Schwab W., Fernie A.R., Muñoz-Blanco J., Molina-Hidalgo F.J. (2022). Strawberry Fruit *FanCXE1* Carboxylesterase Is Involved in the Catabolism of Volatile Esters during the Ripening Process. Hortic. Res..

[20] Cao X., Su Y., Zhao T., Zhang Y., Cheng B., Xie K., Yu M., Allan A., Klee H., Chen K. (2024). Multi-Omics Analysis Unravels Chemical Roadmap and Genetic Basis for Peach Fruit Aroma Improvement. Cell Rep..

[21] Li Z., Wang Z., Wang K., Liu Y., Hong Y., Chen C., Guan X., Chen Q. (2020). Co-Expression Network Analysis Uncovers Key Candidate Genes Related to the Regulation of Volatile Esters Accumulation in Woodland Strawberry. Planta.

[22] Wang H., Ma Y., Li M., Shi L., Zhang S., Wang W., Yang Z. (2018). Volatiles of ripe fruit *Prunus salicina* L. cv. Friar as determined by gas chromatography-mass spectrophotometry as developed during cold storage. Int. J. Food Prop..

[23] Taiti C., Pandolfi C., Caparrotta S., Dei M., Giordani E., Mancuso S., Nencetti V. (2019). Fruit aroma and sensorial characteristics of traditional and innovative Japanese plum (*Prunus salicina* Lindl.) cultivars grown in Italy. Eur. Food Res. Technol..

[24] Hu X., Li D., Ding Y., Zhang Y., Ren C. (2025). Characteristic of volatile flavor compounds in ‘Fengtangli’ plum (*Prunus salicina* Lindl.) were explored based on GC×GC-TOF MS. Front. Nutr..

[25] Zhang Q., Zhu S., Lin X., Peng J., Luo D., Wan X., Zhang Y., Dong X., Ma Y. (2023). Analysis of Volatile Compounds in Different Varieties of Plum Fruits Based on Headspace Solid-Phase Microextraction-Gas Chromatography-Mass Spectrometry Technique. Horticulturae.

[26] Wu M., Du G., Zhang M., Li S., Geng Y., Wang Y., Bai D., Yang S., Zhu G., Li F. (2026). Genome-Wide Identification and Expression Analysis of LOX-HPL-ADH Pathway Genes Contributing to C6 Volatile Diversity in Chinese Plum (*Prunus salicina*). Horticulturae.

[27] El Hadi M.A.M., Zhang F.-J., Wu F.-F., Zhou C.-H., Tao J. (2013). Advances in Fruit Aroma Volatile Research. Molecules.

[28] Lia X., Gao P., Zhang C., Xiao X., Chen C., Song F. (2023). Aroma of Peach Fruit: A Review on Aroma Volatile Compounds and Underlying Regulatory Mechanisms. Int. J. Food Sci. Technol..

[29] Pino J.A., Quijano C.E. (2012). Study of the Volatile Compounds from Plum (*Prunus domestica* L. cv. Horvin) and Estimation of Their Contribution to the Fruit Aroma. Food Sci. Technol..

[30] Feng J., Xi W., Li W., Liu H., Liu X., Lu X. (2015). Volatile Characterization of Major Apricot Cultivars of Southern Xinjiang Region of China. J. Am. Soc. Hortic. Sci..

[31] Liu H., Yu Y., Zou B., Yu Y., Yang J., Xu Y., Chen X., Yang F. (2023). Evaluation of Dynamic Changes and Regularity of Volatile Flavor Compounds for Different Green Plum (*Prunus mume* Sieb. et Zucc) Varieties during the Ripening Process by HS-GC-IMS with PLS-DA. Foods.

[32] Sirangelo T.M., Rogers H.J., Spadafora N.D. (2022). Molecular Investigations of Peach Post-Harvest Ripening Processes and VOC Biosynthesis Pathways: A Review Focused on Integrated Genomic, Transcriptomic, and Metabolomic Approaches. Chem. Proc..

[33] Ma J., Li X., Chu Y., Yue H., Xu Z., Li B., Wu X., Gan J., Jia Y. (2024). Characterization of Changes in Ripening Process of Volatile Apple Compounds Based on HS-SPME-GC-MS Analysis. Agriculture.

[34] Li X., Li G., Wang H., Liu C., Rong C., Song F., Chen C., Wu J. (2025). Characterization of Volatile Flavor Profiles in Three Peach Cultivars during Postharvest Storage at Various Temperatures Using HS-SPME-GC-MS. Food Chem. X.

[35] Kim K., Chun I.-J., Suh J.H., Sung J. (2023). Relationships between Sensory Properties and Metabolomic Profiles of Different Apple Cultivars. Food Chem. X.

[36] Fan Z., Hasing T., Johnson T.S., Garner D.M., Schwieterman M.L., Barbey C.R., Colquhoun T.A., Sims C.A., Resende M.F.R., Whitaker V.M. (2021). Strawberry Sweetness and Consumer Preference Are Enhanced by Specific Volatile Compounds. Hortic. Res..

[37] Zidi K., Kati D.E., Bachir-Bey M., Genva M., Fauconnier M.-L. (2021). Comparative Study of Fig Volatile Compounds Using Headspace Solid-Phase Microextraction-Gas Chromatography/Mass Spectrometry: Effects of Cultivars and Ripening Stages. Front. Plant Sci..

[38] Yue X., Ju Y., Cui Y., Wei S., Xu H., Zhang Z. (2023). Evolution of Green Leaf Volatile Profile and Aroma Potential during the Berry Development in Five *Vitis vinifera* L. Cultivars. Food Chem. X.

[39] Luo M., Zhou X., Sun H., Zhou Q., Ge W., Sun Y., Yao M., Ji S. (2021). Insights into Profiling of Volatile Ester and LOX-Pathway Related Gene Families Accompanying Post-Harvest Ripening of ‘Nanguo’ Pears. Food Chem..

[40] Souleyre E.J.F., Nieuwenhuizen N.J., Wang M.Y., Winz R.A., Matich A.J., Ileperuma N.R., Tang H., Baldwin S.J., Wang T., List B.W. (2022). Alcohol Acyl Transferase Genes at a High-Flavor Intensity Locus Contribute to Ester Biosynthesis in Kiwifruit. Plant Physiol..

[41] Souleyre E.J., Chagné D., Chen X., Tomes S., Turner R.M., Wang M.Y., Maddumage R., Hunt M.B., Winz R.A., Wiedow C. (2014). The *AAT1* Locus Is Critical for the Biosynthesis of Esters Contributing to ‘Ripe Apple’ Flavour in ‘Royal Gala’ and ‘Granny Smith’ Apples. Plant J..

[42] Huang D., Ma F., Wu B., Lv W., Xu Y., Xing W., Chen D., Xu B., Song S. (2022). Genome-Wide Association and Expression Analysis of the Lipoxygenase Gene Family in *Passiflora edulis* Revealing PeLOX4 Might Be Involved in Fruit Ripeness and Ester Formation. Int. J. Mol. Sci..

[43] Li X., Qi L., Zang N., Zhao L., Sun Y., Huang X., Wang H., Yin Z., Wang A. (2022). Integrated metabolome and transcriptome analysis of the regulatory network of volatile ester formation during fruit ripening in pear. Plant Physiol. Biochem..

[44] Cao X., Wei C., Duan W., Gao Y., Kuang J., Liu M., Chen K., Klee H., Zhang B. (2021). Transcriptional and Epigenetic Analysis Reveals that NAC Transcription Factors Regulate Fruit Flavor Ester Biosynthesis. Plant J..

[45] Guo Y.F., Zhang Y.L., Shan W., Cai Y.J., Liang S.M., Chen J.Y. (2018). Identification of Two Transcriptional Activators MabZIP4/5 in Controlling Aroma Biosynthesis Genes during Banana Ripening. J. Agric. Food Chem..

[46] Lin H., Bai L., Wei W., Su W., Wu Y., Wu R., Wang H., Chen J., Fan Z. (2024). The Role of MaWRKY70 in Regulating Lipoxygenase Gene Transcription during Chilling Injury Development in Banana Fruit. Foods.

[47] Duan W., Yang C., Cao X., Wei C., Chen K., Li X., Zhang B. (2023). Chilling-Induced Peach Flavor Loss Is Associated with Expression and DNA Methylation of Functional Genes. J. Adv. Res..

[48] Han X., Zhang Y., Cao C., Xiao C., Lu W., Dong L., Zhu H., Fu J. (2025). Regulatory Role of AdABI5 Transcription Factor in Enhancing Aroma Biosynthesis in Cold-Stored Kiwifruit. LWT.

[49] Qin G., Wang Y., Cao B., Wang W., Tian S. (2012). Unraveling the Regulatory Network of the MADS Box Transcription Factor RIN in Fruit Ripening. Plant J..

[50] Sun H., Lu X., Wang Y., Li J., Liu S. (2024). Study on Evaluation of Fruit Aroma of Plum Variety Resources Based on Headspace Solid-Phase Microextraction Combined with Gas Chromatography-Mass Spectrometry. Foods.

[51] (2023). NIST Standard Reference Database 1A: NIST/EPA/NIH Mass Spectral Library 2023.

[52] Fiol A., García S., Dujak C., Pacheco I., Infante R., Aranzana M.J. (2022). An LTR Retrotransposon in the Promoter of a *PsMYB10.2* Gene Associated with the Regulation of Fruit Flesh Color in Japanese Plum. Hortic. Res..

