# Supplementary figures and images for: PsAAT3 Drives Ester Accumulation and Fruity Aroma Formation During Ripening in Chinese Plum (Prunus salicina) Through Integrated Volatile Profiling and Transcriptomics
Source: Plants (Basel). 2026 Apr 8;15(8):1144. doi: 10.3390/plants15081144 (PMC13119267; doi:10.3390/plants15081144)

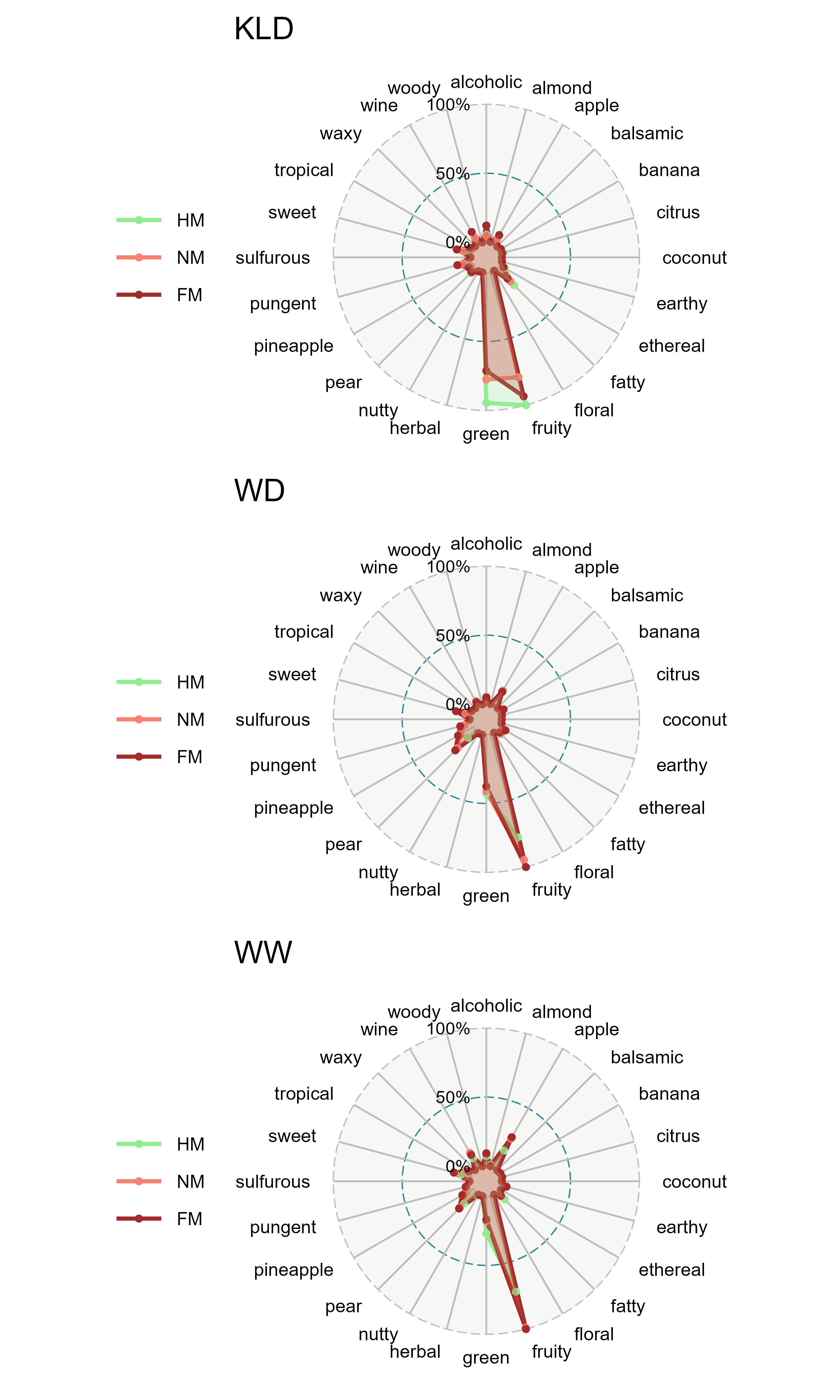

Supplement: Supplementary file 1 [file plants-15-01144-s001.zip › Supplementary Figure S1.png]

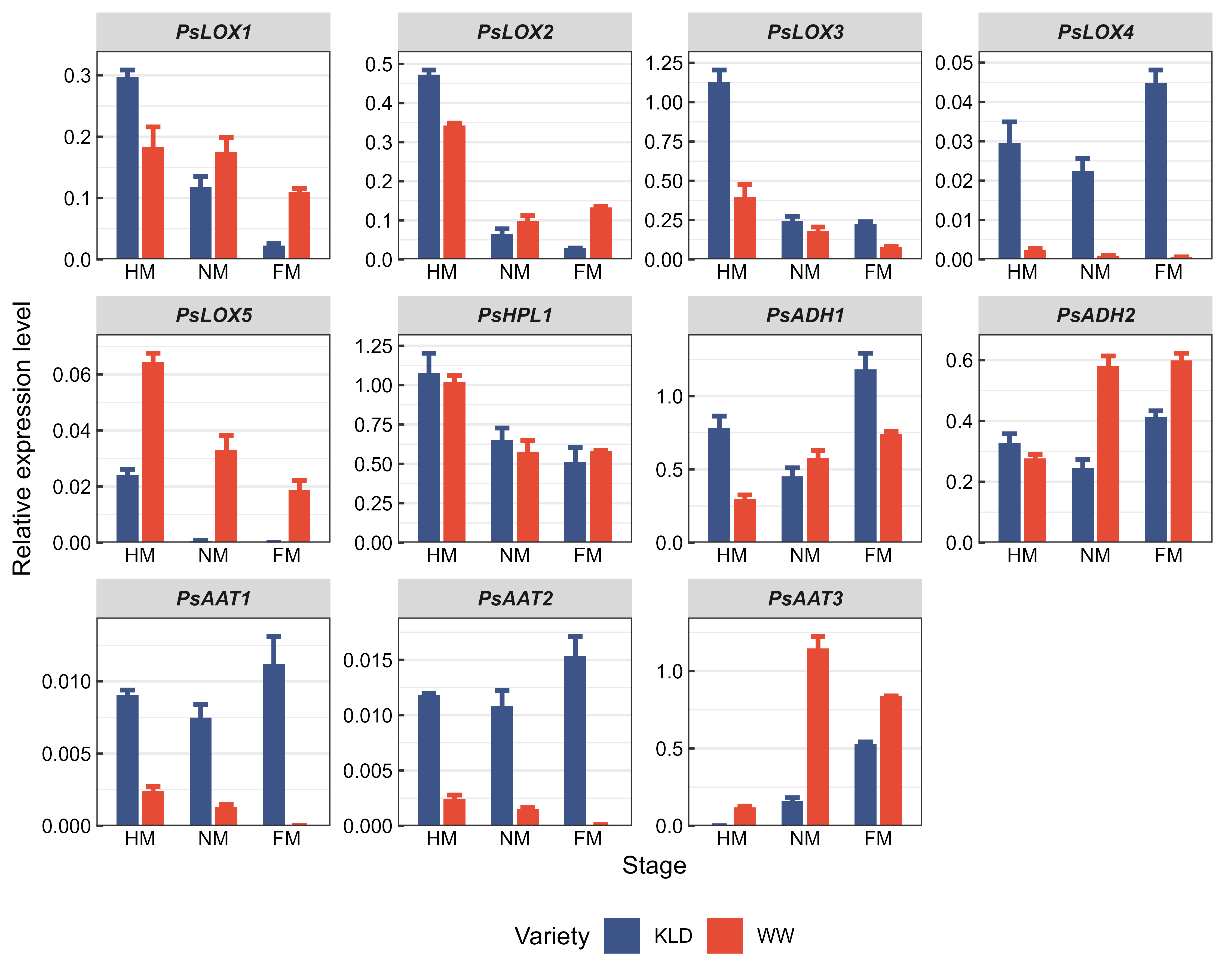

Supplement: Supplementary file 1 [file plants-15-01144-s001.zip › Supplementary Figure S2.png]

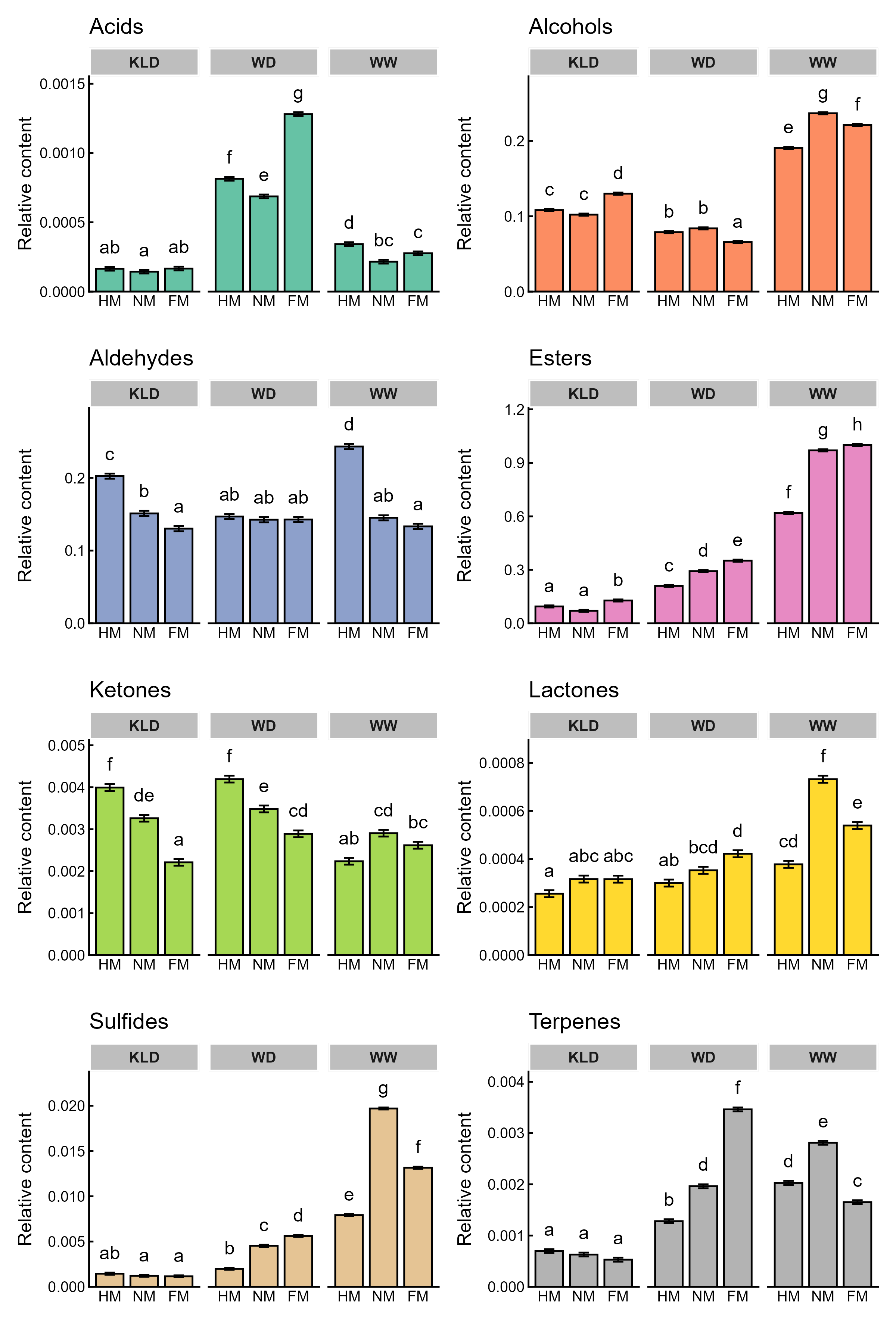

Supplement: Supplementary file 1 [file plants-15-01144-s001.zip › Supplementary Figure S3.png]

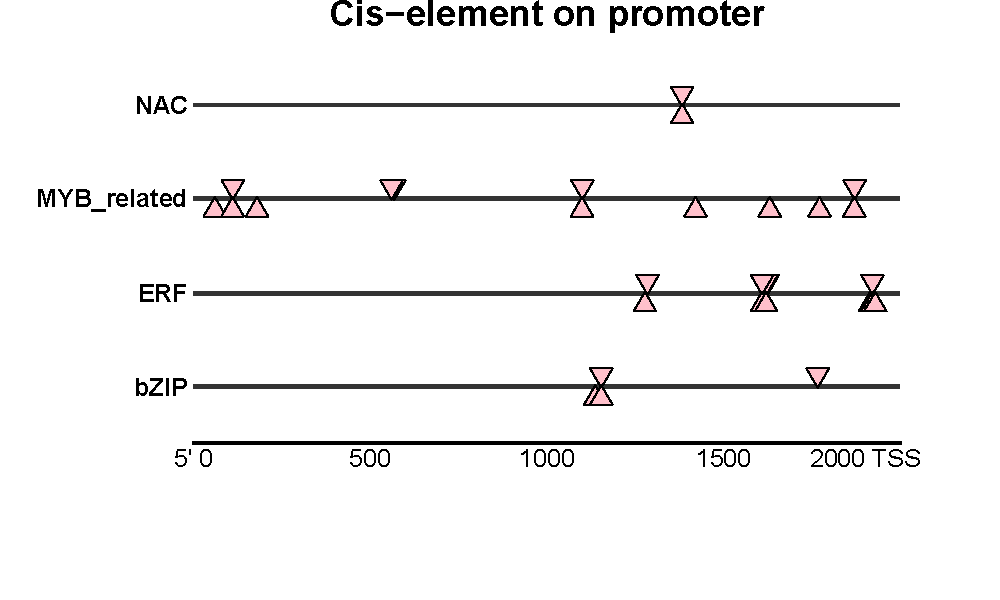

Supplement: Supplementary file 1 [file plants-15-01144-s001.zip › Supplementary Figure S4.png]
